# Corneal Allograft Rejection: Immunopathogenesis to Therapeutics

**DOI:** 10.4172/2155-9899.S9-006

**Published:** 2013-11-20

**Authors:** Yureeda Qazi, Pedram Hamrah

**Affiliations:** 1 Ocular Surface and Imaging Center & Cornea Service Massachusetts Eye and Ear Infirmary, Department of Ophthalmology, Harvard Medical School, Boston, MA, USA; 2 Schepens Eye Research Institute, Massachusetts Eye & Ear, Department of Ophthalmology, Harvard Medical School, Boston, MA, USA

**Keywords:** Immune privilege, Allograft rejection, Antigen presenting cells, Human leukocyte antigen, Angiogenesis, Lymphangiogenesis, Keratoplasty, Immune suppression

## Abstract

Corneal transplantation is among the most successful solid organ transplants. However, despite low rejection rates of grafts in the ‘low-risk’ setting, rejection can be as high as 70% when grafted into ‘high-risk’ recipient beds. Under normal homeostatic conditions, the avascular cornea provides a unique environment that facilitates immune and angiogenic privilege. An imbalance in pro-inflammatory, angiogenic and lymphangiogenic mediators leads to a breakdown in corneal immune privilege with a consequent host response against the donor graft. Recent developments in lamellar and endothelial keratoplasties have reduced the rates of graft rejection even more, while providing improved visual outcomes. The corneal layer against which an immune response is initiated, largely determines reversibility of the acute episode. While epithelial and stromal graft rejection may be treated with topical corticosteroids with higher success, acute endothelial rejection mandates a more aggressive approach to therapy due to the lack of regenerative capacity of this layer. However, current immunosuppressive regimens come with the caveat of ocular and systemic side effects, making prolonged aggressive treatment undesirable. With the advent of biologics, efficacious therapies with a superior side effect profile are on the horizon. In our review we discuss the mediators of ocular immune privilege, the roles of cellular and molecular immune players in graft rejection, with a focus on human leukocyte antigen and antigen presenting cells. Furthermore, we discuss the clinical risk factors for graft rejection and compare rates of rejection in lamellar and endothelial keratoplasties to traditional penetrating keratoplasty. Lastly, we present the current and upcoming measures of therapeutic strategies to manage and treat graft rejection, including an overview of biologics and small molecule therapy.

## Introduction

Corneal transplantation is among the most prevalent and successful organ transplants performed worldwide [1] with more than 65,000 corneas transplanted annually [2]. An overwhelming majority of these surgeries are performed in the United States, summing to 46,684 procedures in 2012 alone [3]. Whilst first-time ‘low-risk’ graft recipients may experience a success rate of 90% in the first two years without a need for systemic immunosuppression or histocompatibility leukocyte antigen (HLA) matching [4,5], up to 70% face graft failure in the ‘high-risk’ setting of vascularized corneal beds or a previous history of graft rejection [6]. In the past decade, there has been a change in surgical trends in corneal transplantation, moving away from full-thickness grafts in all patients to lamellar procedures in cases when specific layers are affected for corneal diseases that differentially affect selective layers of the cornea [7–10]. Penetrating keratoplasty, which is a full-thickness transplant procedure, has been surpassed in its rates of graft survival, rejection reversibility, visual and long-term surgical outcomes by selective lamellar keratoplasties that aim to only treat the affected corneal layer [11–16]. Although the cornea is an immune privileged site, graft rejection may ensue following breakdown of its immune privilege, by corneal neovascularization, inflammation, or trauma to the graft among several other factors discussed herein. In this review we discuss the molecular and cellular perpetrators of immune-mediated graft rejection with emphasis on the roles of corneal dendritic cells (DCs) and the human leukocyte antigens; risk factors than can threaten corneal immune privilege, inducing immune-mediated graft rejection; rejection rates associated with full-thickness and lamellar keratoplasties; and both current and evolving therapeutics in the prophylaxis and management of corneal graft rejection.

## Mechanisms of Corneal Graft Rejection

### Human leukocyte antigen (HLA)

All cells of individuals within a single species express surface polymorphic proteins known as major histocompatibility complex (MHC) antigens, which differentiate one individual from another. MHC genes are prolifically polymorphic with over 200 allelic variants, therefore further increasing the odds of mismatched antigens between donor and host corneas in allogeneic transplants, creating an optimal opportunity for graft rejection; these antigens are thus colloquially termed “major transplantation antigens” [17]. Chromosome 6 bears a dense region of contiguous MHC genes. In humans, the genes which code for these “major transplantation antigens” on chromosome 6 are called human leukocyte antigen (HLA) genes [18]. MHC antigens can be classified into two distinct types. MHC type I (MHC-I) is found on all nucleated cells of the body and platelets. In the cornea, MHC-I antigens are expressed by corneal epithelial, stromal and endothelial cells. In humans, these transmembrane glycoproteins are coded for by HLA-A, HLA-B and HLA-C genes. The other category of MHC antigens is type II (MHC-II). MHC-II antigens are more selectively expressed, and are limited to the cell surface of immunocompetent antigen-presenting cells (APCs) such as DCs, macrophages and Langerhans cells [18,19]. These MHC antigens are expressed by HLA-DQ, -DR and –DW genes in humans and are not constitutively expressed in the center of an otherwise healthy cornea [20]. Foreign antigens are presented to naïve T cells by APCs as processed peptides in the presence of MHC-II and co-stimulatory molecules to program recognition of “non-self” antigens. Inflammation, interferon gamma (IFN-γ) and surgery can induce expression of MHC-II antigens in the cornea.

Corneal DCs play a critical role in graft rejection through their ability to regulate T cell responses to both self and foreign antigens in the donor button, based on molecular cues received from the tissue microenvironment [21]. Antigen recognition and capture occur through MHC class-II antigens on the surface of mature DCs. The majority of resident central corneal DCs reside in a dormant, undifferentiated state, and are MHC-II negative [22]. During an inflammatory insult, DCs traffic from the blood to the cornea where donor alloantigens are captured and transported to secondary lymphoid organs for processing and presentation to T cells, thereby modulating the T cell response.

The evidence on the benefit of class I HLA tissue matching in reducing allogeneic corneal graft rejection is fairly conclusive with exclusion of the Collaborative Corneal Transplantation Studies (CCTS) data, demonstrating a clear benefit of prolonged graft survival in type I HLA-matched corneal transplant cases [5,23,24]. Unlike the defined advantage of type I HLA matching, a consensus on the utility of type II HLA matching remains controversial. HLA-DR matching between donor and recipient has generated a spectrum of conflicting data ranging from facilitating a prolonged graft survival rate of 79% in HLA-DR matched high-risk eyes [25], to no observable effects as reported by the CCTS study and studies conducted by Japanese groups [5], and even proving detrimental to the corneal button in some populations [26,27]. To date, the only randomized, double-blinded, prospective, controlled clinical trial evaluating donor-recipient histocompatibility matching and cross-matching on graft survival in high-risk corneal transplantation is the CCTS [5]. The study however failed to detect any beneficial effect of tissue matching on the rate of graft failure, rate of graft rejections, or the rate of failure caused by rejection [5]. The two main criticisms of the CCTS are: first, that it included patients with limbal stem cell deficiency, which could have masked the beneficial effect of HLA matching, and second, that it employed an intense steroid regimen that could have suppressed HLA expression.

Murine studies have provided a sound platform to advocate the utility of tissue matching in human corneal transplantation to prevent sensitization of the host to donor MHC antigens leading to reduced incidence of graft rejection [23]. HLA matching in a controlled setting among normal-risk patients demonstrated a clear advantage in ensuring clear and rejection-free corneas [24]. Despite the controversy regarding the therapeutic benefit of HLA matching in preventing corneal graft rejection [5,28], several reports suggest an association between HLA incompatibility and graft rejection, and consequently the advantage of HLA tissue typing especially in high-risk patients [28–32]. It has been shown that actively rejecting grafts are strongly associated with primed, donor HLA-class I-specific cytotoxic T cells, making a strong case in favor of HLA-A and –B typing for high-risk transplant patients [29,33–36]. Indeed, the greater the number of HLA-A and HLA-B mismatches, the greater the risk of graft rejection [36–39].

Another class of cell-surface proteins are the minor histocompatibility antigens, which unlike the MHC, are coded for throughout the genome at various loci. In order to be recognized as non-self antigens, minor histocompatibility antigens must be processed by host APCs and presented with MHC-II molecules. ABO blood antigens are a relevant example of such antigens and found on corneal epithelial cells whose expression is increased in graft rejection. The CCTS proposed that ABO matching may reduce the risk of allogeneic graft rejection in high-risk corneal transplantation [5]. It also reported that ABO incompatibility increased the adjusted risk of failure from any cause and failure from rejection [26], with estimated rejection rates of 41% in the ABO incompatible group compared to 31% in the compatible group. These findings are consistent with other studies that indicate non-MHC antigens may play an important role in corneal allograft rejection [26,40], and the fact that ABO blood group antigens are expressed by the corneal epithelium as well [41]. The potential disadvantages of tissue matching in corneal transplantation however do merit a discussion. ABO matching is relatively inexpensive, and about 70% of donors and recipient pairs would be compatible by chance. In contrast, HLA matching may be logistically more complicated; along with the long duration of finding a suitable donor, there may be a significant economic burden associated with obtaining HLA-matched donor tissues for high-risk patients, totaling to an additional USD 4 million per annum [26]. According to a mathematical model, waiting times were predicted to be 15 months for a zero mismatch, and 9 months for a single HLA mismatch [36,42]. Overall, there appears to be considerable benefit to tissue matching, especially in high-risk transplantation. In the wake of the CCTS trial, a more carefully designed clinical trial may provide conclusive answers to this query.

### Corneal immune privilege

The concept of corneal immune privilege is entertained by observations of high survival rates of allogeneic corneal grafts despite HLA mismatching between donor and host tissues. It is the uniquely configured corneal anatomy and physiology of the anterior chamber that evade a host immune response through low immunogenicity and generation of alloantigen tolerance [18,19,43]. The cornea is a uniquely avascular tissue and free of lymphatics, preventing direct access of the immune system to the cornea through lack of vasculature, and barring free transport of antigens and APCs to T cell-rich secondary lymphoid organs through absence of lymph vessels. Further, all layers of the cornea have low constitutive expression of MHC-I and –II antigens, limiting immunogenicity to foreign antigens. Even though DCs are present both in the central and peripheral cornea, they exist in an immature, inactivated state, maintaining immune quiescence in a healthy cornea. The cornea expresses many cell membrane-bound molecules that guard the cornea from immune-mediated inflammation and induce apoptosis of immune effector cells. These molecules include complement regulatory proteins (CRP), Fas ligand (FasL), MHC-Ib and tumor necrosis factor (TNF)-related apoptosis-inducing ligand (TRAIL). FasL (CD95L), a pro-apoptotic molecule, is expressed by the corneal epithelium and endothelium. FasL serves to destroy polymorphonuclear neutrophils (PMNs) and effector T cells that express its receptor Fas/CD95, promoting immune quiescence while protecting against immune-mediated graft rejection [44,45]. The corneal epithelium, stroma and cells of the ciliary body also express programmed death ligand-1 (PD-L1), which upon interaction with its cognate receptor (PD-1) on T cells leads to inhibition of T cell proliferative capacity, induction of apoptosis and suppression of IFN-γ secretion [46], promoting graft survival [47,48]. Expression of PD-1 by T cells is regulated by Notch signaling [49]. PD-1 inhibits T cell proliferation though suppression of Ras and Akt signaling pathways which inhibit transcription of SKP2 leading to upregulation of transforming growth factor-beta (TGF-β)-specific transcription factor Smad3, resulting in cell cycle arrest of T cells [50].

The anterior chamber is rich in soluble immunosuppressive factors such as TGF-β, alpha-melanocyte stimulating hormone (α-MSH), calcitonin gene-related peptide (CGRP), CRP, somatostatin (SOM), indoleamine dioxygenase (IDO), vasointestinal peptide (VIP) and macrophage migration inhibitory factor (MIF), which inhibit T cell and complement activation [18,51]. The most notable contribution is that of anterior chamber-associated immune deviation (ACAID), an alloantigen-specific peripheral immune tolerance to antigens in the anterior chamber, capable of deviating the systemic cytotoxic immune response [52,53]. ACAID suppresses delayed-type hypersensitivity (DTH) response and maintains humoral immunity, promoting graft survival [54,55]. Antigens within the anterior chamber are recognized and processed by F4/80^+^ APCs that orchestrate allotolerance by upregulating the expression of TGF-β with downregulation of the co-stimulatory molecule CD40/CD40L and interleukin-12 (IL-12) [53,56,57]. Suppression of DTH is brought about by migration of these APCs to the spleen through vascular elements in the trabecular meshwork, and together with splenic accessory immune cells, alloantigen-specific tolerance is achieved [58–60]. While the effect of ACAID on DTH is unequivocal, its impact on the regulation of cytotoxic T lymphocytes (CTL) is more complex, being dictated by the nature of the antigens present in the anterior chamber. Some groups have demonstrated that CTL function remains intact through expression of CTL precursors and effectors in the spleen and lymph nodes of animals inoculated with tumor cells *in vitro* [60,61], making the hypothesis of a suppressed CTL response an unlikely explanation for tumor growth in ACAID. In contrast, other groups that used a soluble antigen for intracameral inoculation observed inhibition of antigen-specific CD8^+^ T cell responses, confirming the antigen-dependent effect of ACAID on CTL [62–66]. Since CTL responses contribute to allogeneic corneal graft rejection even though they are not known to be directed against MHC alloantigens [67–70], the effect of ACAID on CTL function against MHC antigens and the involvement of FoxP3^+^ regulatory T cells (Treg) in modulating CD8^+^ T cell function during ACAID have been explored [71]. Both CD4^+^ T and CD8^+^ T cell populations in the spleen proliferate upon MHC alloantigen-specific ACAID induction, however, once ACAID is expressed, the percentages of these T cells decrease substantially, suggesting ACAID-mediated inhibition of both CD4^+^ T and CD8^+^ T cell function. Therefore, we now know that solubility of the antigen is not a necessary determinant of ACAID-mediated CTL immune suppression. Therefore measures to promote ACAID-mediated inhibition of DTH and CTL could prove beneficial in prolonging graft survival. Interestingly, while FoxP3^+^ regulatory T cells (Treg) increase upon ACAID induction, they have not been shown to be directly involved in the modulation of ACAID-mediated MHC alloantigen-specific T cell function and response [71].

### Immunology of Corneal Graft Rejection

Corneal graft rejection occurs when the host immune response is directed toward antigens in the donor corneal button, leading to tissue destruction brought about by cells and mediators of the innate and adaptive immune responses. An immune response may target any of the main layers of the cornea selectively, or, in combination. Compromise of the corneal epithelium and stroma may be reversible, but, rejection of the endothelium invariably results in irreversible endothelial cells loss and may result in permanent graft failure, if not treated judiciously [72]. Sensitization of the host to donor antigens forms the “afferent” arm, also known as the induction phase of corneal allograft rejection. This allorecognition process is orchestrated by APCs presenting donor antigens to naïve T cells in draining lymph nodes in either a direct or indirect fashion [18]. The *direct pathway* constitutes presentation of donor antigens to naïve T cells directly by *donor* APCs through non-self MHC-II recognition on their surface, resulting in proliferation of direct alloreactive T effector cells [19]. In contrast, the *indirect pathway* yields donor antigens to *host* APCs that travel the cornea, capture donor antigens, and transport them to draining lymph nodes where antigen presentation occurs through recognition of self MHC-II by naïve T cells [19]. While initially believed to be a phenomenon brought about exclusively by the *indirect pathway* [67], accumulated evidence indicates that both the *direct* and *indirect pathways* are implicated in the immune-mediated rejection of orthotopic corneal allografts, especially in high-risk corneal beds with higher immunogenicity and compromised immune privilege [73–78]. The cornea harbors resident populations of the most potent bone marrow-derived epithelial and stromal APCs [22,79], namely, DCs, which are pivotal to the modulation of corneal immunogenicity [80]. These resident DCs are uniformly immature and MHC-II low/negative in the corneal center, but with a change in the microenvironment of the cornea from a quiescent to an inflammatory state, as in corneal transplantation, they express MHC-II and other co-stimulatory molecules, as well as increase in density [22,79,81,82]. More recently additional subpopulations of corneal DCs have been identified, adding to the complexity of the corneal immune system [83–86]. Once DCs undergo maturation, they express co-stimulatory molecules such as CD80, CD86 and CD40 [81], as well as differential adhesion molecules, that activate T cell receptors and induce T cell proliferation through concurrent release of cytokines. Among such cytokines are IL-1, -6 and -12 released by the APCs [80].

Lymph nodes serve as the priming hub for T cell allosensitization and activation, which then drives the subsequent “efferent” arm, or the expression phase, of immune-mediated graft rejection. It is this phase that results in the actual destruction of the graft, making lymph nodes profoundly critical to the process of rejection [87]. In support of the importance of draining lymph nodes in the rejection process, several murine studies have demonstrated that cervical lymphadenectomy prior to orthotopic corneal transplantation yields near complete graft acceptance along with suppressed allospecific DTH response, regardless of the pre-operative risk [77,88]. Following sensitization and activation of naïve T cells, cytokines and chemokines released induce proliferation and trafficking of these alloreactive T cells to the cornea through expression of specific combinations of adhesion molecules [89]. Chemokines (*chemo*tactic cyto*kines*), are small-molecule-weight cytokines that modulate recruitment of leukocytes and immune cells to the inflamed cornea [80,89]. Immune-mediated damage to the graft begins with the release of cytokines, such as tumor necrosis factor-alpha (TNF-α) and IL-1, secondary to the mechanical trauma of surgery. In the setting of high-risk corneal transplantation, cytokines further induce the production of various early chemokines. Overexpression of chemokines monocyte chemotactic protein-1 (MCP-1), chemokine C-C motif ligand 2 (CCL2), regulated on activation normal T cell expressed and secreted (RANTES; CCL5), macrophage inflammatory protein (MIP), MIP-1α (CCL3) and MIP-1β (CCL4) in acute graft rejection leads to additional recruitment of APCs and T cells into the cornea [18,90–92].

Once the graft and infiltrating leukocytes release late chemokines, guidance of alloreactive T cells towards the graft begins [19,93]. Alloreactive T cells then migrate to the cornea where they recognize donor MHC antigens, and also induce the development of memory T cells so that an immune response may be mounted against the same antigens upon re-exposure as in the case of a re-graft [19]. The primary cellular mediators of graft rejection are CD8^+^ CTL, and CD4^+^ T-helper (Th) lymphocytes, otherwise known as DTH cells. Even though the role of CTL in corneal graft rejection remains somewhat controversial, they are believed to be sufficient but not necessary for corneal graft rejection [69,94]. Based on the types of cytokines secreted by Th lymphocytes, T cells can be further classified into Th1, Th2 and the more recently discovered Th17 cells [95,96]. Th1 cells are largely considered to be the primary effector cells in corneal graft rejection [19,97]. CD4^+^ Th1 cells secrete IL-2, IFN-gamma and lymphotoxin, which lead to inflammation as an attack on the inciting antigen. IL-2 is critical to a sustained immune response by its positive feedback on T and B cell activation and proliferation. IFN-γ ensures that macrophages are activated at the site of inflammation, and facilitates further expression of MHC-II antigens in the donor button. Th17 cells on the other hand secrete IL-17, IL-21 and IL-22 [98]. TGF-β is the key differentiation factor for Th17 cells, which acts in concert with IL-6 or IL-21 and IL-23 serves as a stabilizing factor for maintaining the Th17 lineage [99–103]. IL-1 signaling also comes into play via IL-1β-mediated regulation of the dendritic cell-mediated Th17 cell differentiation pathways and maintenance of cytokine expression in Th17 cells [104]. Interestingly, TGF-β is also an inducer of CD4^+^CD25^+^Foxp3^+^ Tregs [105]. Therefore, generation of Th17 cells or CD4^+^CD25^+^Foxp3^+^ Tregs is largely dictated by the cytokine milieu of the tissue microenvironment [99,106,107]. Murine studies demonstrate increased expression of Th17 cells in the early stages of corneal allograft rejection followed by predominance of a Th1 response in the late stage [108]. However, Th17 do not orchestrate the corneal immune response in graft rejection independently. The role of IL-17 in graft rejection is somewhat limited. While some studies using monoclonal antibodies against IL-17 successfully demonstrate a moderate increase in murine corneal allograft survival [109], IL-17 knockout studies in mice failed to show improved graft survival [108]. This has been postulated to be as a result of an emerging Th2 response, which then mediates graft rejection [109,110]. However, there still remains controversy regarding clearly defined pathways given the complex interplay of immune cells in corneal allograft rejection. Murine IFN-γ and IL-17 knockout studies have shown that even MHC-matched corneal allografts are rejected in an IFN-γ- and IL-17-independent manner, suggesting mediators other than the simplistic model of Th1, Th2 or Th17 pathways [111].

### Risk Factors of Corneal Graft Rejection

Both host and donor factors may contribute to the development of immune-mediated graft rejection. Among the most widely accepted and well-established risk factors for corneal allograft rejection are: presence of stromal blood vessels in one or more quadrants of the recipient cornea (high risk), with at least a 6 clock-hour distance between the two blood vessels, corneal neovascularization after surgery, prior graft rejection episodes, pre-operative glaucoma, young age, prior anterior segment surgery, active ocular inflammation, ocular surface disease, herpes simplex keratitis, neurotrophic keratopathy, large and eccentric grafts, and anterior synechiae [6,112–114].

## Corneal Allograft Rejection

### High-risk

A high-risk cornea is defined by the CCTS as one with two or more quadrants of deep stromal vessels prior to surgery, or, one in which a prior graft has been rejected [5]. Presence of stromal vascularization in all four corneal quadrants doubles the risk of rejection [5], increases the severity of immune response against the graft [115–117], and reduces the time taken to reject the graft [118]. Rejection rates increase from 14% in avascular or minimally vascular corneas to 32% in the presence of preoperative stromal neovascularization [119]. The 2-year survival of grafts placed in high-risk corneas is less than 50% [1]. History of a prior episode of immune-mediated graft rejection poses a significant threat to the new graft. The CCTS reported a cumulative increase in the risk of rejection by a factor of 1.2 with every successive re-graft [5]. Taking into account and normalizing for corneal neovascularization, patients may have rejection rates of 40%, 68% and 80% after the first, second and third re-grafts [118].

### Low-risk

Based on the number of corneal quadrants with stromal vascularization, a low-risk transplant is defined as a graft placed in a recipient bed that is avascular. The 2-year survival rate associated with low-risk grafts is an impressive near 90% [1,120,121], maintaining survival rates of 90% and 82% at 5 and 10 years post-transplant [122,123].

### Endothelial, lamellar and penetrating keratoplasties

With the recent development of more sophisticated sutureless surgical techniques that dissect and replace the selected layer of diseased cornea, lamellar and endothelial keratoplasties have led to reduction in graft rejection rates and improved surgical and visual outcomes [16,124].

#### Endothelial keratoplasty

Descemet’s stripping automated endothelial keratoplasty (DSAEK) and Descemet’s membrane endothelial keratoplasty (DMEK) allow selective removal of the diseased Descemet’s membrane and endothelium, without disturbing the epithelium and much of the stroma [10,15]. This translates into shorter healing time, lower graft rejection rates, and improved visual quality. In comparison with deep anterior lamellar keratoplasty (DALK) and traditional, full-thickness penetrating keratoplasty (PK), patients that undergo DSAEK report fewer anterior corneal higher-order aberrations (HOA) but lower visual acuity [16,125]. DSAEK is especially beneficial for endothelial diseases such as Fuchs’ endothelial corneal dystrophy (FECD) and pseudophakic bullous keratopathy (PBK), where the disease may be limited to a single corneal layer and employing PKP for a full-thickness graft is to invite unnecessary complications and prolong healing. Patients with FECD suffer from low contrast sensitivity, and even in cases where DSAEK does not objectively improve visual acuity, 88% of these patients had stark improvement in contrast sensitivity (CS), which translated into improved perceived visual quality [126]. The presentation of endothelial rejection in DSAEK is atypical in that a Khodadoust line is rarely seen [127,128]. Due to the absence of donor epithelium and stroma, and lack of direct contact of the endothelium with limbal vessels, DSAEK offers a low endothelial rejection rate of 10%, and is usually responsive to topical steroids [129].

DMEK, a procedure that involves removal and replacement of the Descemet’s membrane and endothelium has generally a better visual acuity outcome than DSAEK [15]. In addition to improved visual acuity, DMEK offers lower endothelial immune reactions [127]. However, preparation of the donor button and attachment pose significant technical issues in the practice of DMEK [15]. Although DMEK has clearly been shown to have long-term graft survival in comparison to DSEK and PKP, a recent clinical report has shown no statistically significant difference in the long-term graft survival rates between DSEK and PKP [127].

#### Lamellar keratoplasty

A preferred procedure for stromal diseases with a healthy endothelium is a technique called deep anterior lamellar keratoplasty (DALK), where the stroma and epithelium are replaced leaving the host endothelium intact [2]. DALK boasts a 10-year stable graft survival rate of 99.3% with an 11% loss in endothelial cell density from pre-operative levels [130]. In randomized clinical trials, DALK emerged superior to PKP in its safety demonstrated through preserved endothelial cell density and normal intraocular pressure, and comparable in visual function outcomes for visual acuity [11,131]. The reported three-year cumulative incidence of irreversible rejection is 0% with DALK grafts, and 5.2% in PKP, whereas the 3-year cumulative rate of a rejection episode is 10% in DALK and 23% in PKP, further emphasizing superiority of DALK over PKP [132].

## Therapies for Corneal Graft Rejection

### Immunosuppressives

#### Topical corticosteroids

Topical corticosteroids are the mainstay of treatment in the management of acute graft rejection and as post-operative prophylactic therapy for high-risk transplant patients. Topical 1% prednisolone acetate is typically prescribed in acute graft rejection. A commonly prescribed regimen involves 1% prednisolone acetate every 1–2 hours for the first few weeks with gradual tapering over several months. Surgeons may maintain indefinite treatment with low-dose steroids [133]. For non-endothelial graft rejection, topical steroids are advised up to six times a day, with tapered dosing over 6–8 weeks. For mild endothelial rejection, the frequency is increased to hourly, along with application of a steroid ointment at night for 3 days, with tapered dosing over a week. However, in severe endothelial rejection topical steroids are prescribed hourly in combination with systemic steroid therapy. Topical steroid use may be advised indefinitely, especially in high-risk patients [134]. As a result of continued corticosteroid use, these patients usually suffer from cataract, glaucoma, impaired wound healing and become prone to infectious keratitis. Synthetic analogues of corticosteroids such as 0.5% loteprednol etabonate suspension may be a viable alternative for patients in need of indefinite immunesuppression due to a safer profile in maintaining lower intraocular pressures [135].

#### Systemic corticosteroids

In cases of severe endothelial graft rejection that present early, there is debate over the benefit of oral systemic steroids versus pulse therapy. In a randomized clinical trial that evaluated the outcomes of topical corticosteroid therapy with either 60 to 80 mg of oral methylprednisolone daily, or, a single intravenous pulse of 500 mg methylpredinosolone, it was observed that pulse therapy was superior oral methylpredinoslone. While the graft survival rates were comparable between the two groups, only 26.3% of the surviving grafts in the pulse therapy group went on to have a second rejection episode in comparison to 66.7% of the grafts in the oral steroid group, with added benefit to repeated pulse therapy [136,137]. Methylprednisolone pulse therapy may be substituted with subconjunctival or posterior subtenon’s triamcinolone injections in combination with topical steroids in the treatment of endothelial rejection [134,138,139].

Alternatively, intravenous dexamethasone, at a dose of 1 mg/kg in patients with severe endothelial rejection can be used for severe rejection. Reversibility of graft rejection is reported to be better with intravenous dexamethasone (72.3% vs. 45%) as observed in a retrospective study comparing combination therapy of topical steroids with either dexamethasone, or, methylprednisolone [140]. Chronic systemic corticosteroid use is associated with complications such as osteoporosis, diabetes, and weight gain. Unless the patient has co-morbid inflammatory disease, long-term oral corticosteroids in the absence of topical immune suppression is not justified and neither does it render protective effects towards graft survival.

#### Calcineurin inhibitors

Cyclosporine A (CsA) and tacrolimus have proven efficacious to varying degrees in the treatment of graft rejection and management of high-risk patients post-transplantation. They provide viable options for sustained immunosuppression as djuvants to corticosteroids. Several groups have investigated the benefit of combination therapy with topical steroids and topical CsA in the management of acute graft rejection and as prophylactic therapy in high-risk patients following keratoplasty. The results of such studies are mixed and controversial. A randomized controlled trial failed to demonstrate any additional benefit of adding 0.05% topical CsA to 1% topical methylprednisolone acetate in the treatment of acute endothelial graft rejection [141]. This study however, not only included patients that were not truly high-risk, but also may have masked any beneficial effect of topical CsA by using a remarkably high frequency dosing protocol of methylprednisolone. On the other hand, case-control studies in a pediatric population noted a perceivable advantage in rejection-free graft survival rate using 2% CsA in addition to topical steroids than steroids alone (88.9% vs. 38.5%, p=0.046) in the management of post-transplant rejection prophylaxis with the caveat that there was no long-term graft survival benefit between the two groups [142]. In other reports, 2% topical CsA ensured graft clarity for up to 16 months [143]. Systemic administration of CsA has proved more effective in the management of high-risk patients after transplantation [144–146], than treating acute graft rejection, with due consideration to be given to patient selection given the serious systemic side effect profile of CsA therapy [147–149]. For patients in whom corticosteroids are contraindicated, topical CsA (0.5%) may provide an efficacious alternative for post-transplant management with maintenance of graft clarity in 88% of the patients, with a 12% rejection rate [150].

Tacrolimus (FK-506), also a calineurin inhibitor and thus similar in its mechanisms of action to CsA, can be used topically or systemically in the management of low- and high-risk corneal transplantation. Randomized clinical trials have demonstrated that low-dose (0.06%) topical tacrolimus at a frequency of thrice daily for 6 months proved superior to topical steroids in preventing rejection episodes (100% vs. 84%) in low-risk patients following corneal transplantation [151]. Likewise, a low dose of 0.03% tacrolimus ointment proved sufficient for the reversal of acute graft rejection episodes in high-risk patients and maintained clear grafts throughout the duration of therapy [152]. Systemic administration of tacrolimus (2–12mg daily) is also beneficial in preventing and treating graft rejection in high-risk patients post-procedure, with a clear graft survival of 65% [153,154].

#### Antimetabolites

Mycophenolate mofetil (MMF), an inhibitor of inosine monophosphate dehydrogenase (IMPDH), inhibits the de novo synthesis of DNA in T and B lymphocytes, making these immune cells cytostatic. Randomized clinical trials testing MMF have demonstrated that the efficacy of MMF in preventing graft rejection in high-risk patients is comparable to that of CsA [155,156]. MMF can also be supplemented with topical steroids in the prevention graft rejection among high-risk patients. The combination of MMF with topical steroids proved superior to CsA with topical steroids in terms of the 3-year rejection-free graft survival (72% vs. 60%, p=0.03) with clear graft survival (87% vs. 77%) and necessitated shorter duration of therapy [144].

### Current novel therapeutics

#### Angiogenesis and lymphangiogenesis

Corneal neovascularization is the strongest determinant of graft rejection prior to transplantation and poses a challenge to reinstating ocular immune privilege in the wake of corneal transplantation [157]. Patent vasculature invites immune and inflammatory cells to the cornea, allowing for immune-mediated interactions with the donor antigens, setting a stage for impending graft rejection. Mechanical treatments to the blood vessels such as photoablation, diathermy and cryotherapy require multiple visits and the results are temporary [158,159]. Given the pivotal role of corneal avascularity in promoting graft survival, optimizing corneal transparency is essential. Vascular endothelial growth factor (VEGF)-A and –C are potent mediators of heme- and lymphangiogenesis, both of which are critical to maintaining corneal transparency. Corneal avascularity is maintained by soluble VEGFR-1, by a VEGF trap mechanism [160]. Suture-induced inflammation induces parallel growth of blood and lymph vessels into the cornea (lymphangiogenesis) [161,162], thereby creating a free-flow channel for the recruitment and migration of primed T cells into the graft, making it imperative to address corneal heme- and lymphangiogenesis. While mechanical treatment of neovessels requires repeated visits, delivery of molecular anti-angiogenic agents can sustainably prime the cornea before transplantation and also regress post-transplantation neovascularization. Subconjunctival injections of bevacizumab, a monoclonal anti-VEGF antibody, have been shown to not only increase graft survival when delivered prior to transplantation, but also regress blood and lymph vessels during graft rejection [163–168]. More recently, murine studies have proved the efficacy of sunitinib, a multi-target tyrosine kinase inhibitor, in the regression of corneal heme- and lymphangiogenesis through inhibition of VEGF-A, VEGF-C and F4/80^+^ cell recruitment [169]. Antisesnse technology has recently attracted attention with the application of morpholine oligonucleotides (morpholinos) to silence or upregulate genes of interest. Morpholinos that induce expression of the soluble form of VEGFR-1 have successfully demonstrated increased graft survival in rodent models following subconjunctival delivery resulting in inhibition of both heme- and lymphangiogenesis [170].

#### Immune cell trafficking

Adhesion molecules mediate trafficking of immune cells to from the lymph nodes towards sites of inflammation such as the graft. Animal studies of transplant rejection among various conditions of allodisparity and expression of intercellular adhesion molecule 1 (ICAM-1) in transgenic mouse models revealed that deficiency of ICAM-1 lead to significant improvement in graft survival in MHC-mismatched grafts, suggesting the role of ICAM-1 in allosensitization of T cells to MHC antigens [171]. Other promising targets for inhibition of corneal graft rejection are leukocyte function antigen-1 (LFA-1) and very late antigen-1 and 4 (VLA-1, VLA-4) [172,173].

#### Lymphocytes

Rapamycin (sirolimus), an inhibitor of a serine-threonine protein kinase, mammalian target of rapamycin complex-1 (mTORC1), inhibitis effector T cell proliferation and activation without compromising the function of regulatory T cells. In an open-label study, rapamycin has been shown to have a 1-year rejection prevention rate of 78% and was comparable in its efficacy to MMF in the management of high-risk transplant patients [174]. MMF and rapamycin may also be used in combination to prolong corneal graft survival in high-risk patients [175], however, caution should be exercised before administering this drug, alone or in combination with other immunosuppressives given its systemic toxicity profile.

Another evolving approach to regulating effector T cell response is by delivering monoclonal antibodies against T cell antigens. Monoclonal antibodies to CD3 and CD6 can successfully treat episodes of acute graft rejection when delivered intracamerally [176,177]. Daclizumab and basiliximab both target the IL-2 receptor expressed on activated T cells to inhibit T cell proliferation. Monoclonal antibodies may be combined with immunosuppressives such as CsA in the management of high-risk transplant patients. One such study investigated the utility of basiliximab and CsA in high-risk keratoplasty patients. None of the patients suffered immune-mediated rejection edpisodes and maintained clear grafts for up to a duration of 25 months [178]. When used as monotherapy, basiliximab shows lower efficacy than CsA in preventing and treating graft rejection episodes following high-risk keratoplasty. Patients that received basiliximab had a 40% rejection rate, and 50% of the rejected grafts lead to graft failure [179]. Nevertheless, in terms of safety, none of the patients developed side effects with use of this monoclonal antibody.

#### Co-Stimulatory blockade

Activated T cells are critical to immune-mediated graft destruction. Complete and effective activation of T cells is contingent upon binding of T cell receptor/CD3 with MHC molecules on APCs, and interaction of T cell co-stimulatory molecule CD28 with B7 antigens on APCs (CD80 and CD86) [180]. Cytotoxic T-Lymphocyte Antigen 4 protein (CTLA4-Ig), a fusion protein that competitively inhibits the binding of B7 antigens with CD28, inhibits the activation of T cells and has been shown to prolong corneal allograft survival after systemic injections or gene delivery to the corneal epithelium [181,182]. Systemic administration of anti-CD28 monoclonal antibody or local administration of non-functional CTLA4-Ig in rats, has also demonstrated prolonged graft survival [183]. Moreover, incubation of rabbit donor cornea with CTLA4-Ig, prior to grafting, enhances allograft survival in high-risk but not low-risk corneal transplantation [184]. Furthermore, blockade of CD40–CD154 pathway by the use of anti-CD 154 monoclonal antibody has been shown to prevent corneal allograft rejection and promote the acceptance in 100% of low-risk and in over 90% of high-risk grafts after systemic injections, and to a lesser degree with topical use [185,186]. The role of both host and donor PD-L1 has recently been demonstrated in corneal transplantation [47], blockade of which led to enhanced graft survival [187]. Given the promise of localized, targeted therapy with a safe side effect profile, treatment with biologics is promising for the future management of corneal graft rejection.

## Summary and Future Directions

The pathophysiology of corneal allograft rejection is highly complex and still, in part, elusive. Nevertheless, significant advances have been made over the past several decades in our understanding of the mechanisms of corneal graft rejection. Investigations probing into the mechanisms and dissecting immune cell signaling and trafficking pathways are needed to further unravel the cellular and molecular mediators in corneal graft rejection. Development of targeted biological therapy in the future may provide new avenues in the treatment of corneal graft rejection with much safer side effect profiles and sustained therapeutic effects at lower doses. Further, the prospect of modulating immune cell activity through the use of morpholine oligonucleoitides, RNA interference and targeted microRNAs could be promising toward immunotherapy with minimal off-target effects. With the development of new cellular and molecular therapeutic approaches, meticulous clinical trials may then be designed to test their clinical utility. Currently, there is paucity of randomized controlled trials in corneal transplantation research, making conclusions about the safety and efficacy of treatment regimens limited.
